# Event and Comment

**Published:** 1912-02-15

**Authors:** 


### DENTAL REGISTER.

**Vol. LXVI. February 15, 1912. No. 2**

### EVENT AND COMMENT.

THE PASSING OF MEN OF MARK. In the last days of the past year the dental profession has lost some men who helped materially to make strong the position of our calling as a learned and artistic profession. Dr. S. G. Perry of New York was a rare man in many ways. He was not only and always the courteous and well bred gentleman, but, by his dignified maner and rare technical skill, he was a man of wide reputation for all the higher qualities and ideals which go to make up the true professional man.

Dr. C. M. Wright, who was also so suddenly called away from us was a typical professional man, who left his impress on the profession. As a man, friend and teacher he had rare gifts which gave him a social and professional power, which he never neglected to use in behalf of the cause which was so dear to his heart. Wright like Perry had hosts of friends, and they all felt that he had a special interest in them, which he undoubtedly did, as he loved all good men, and he did it in such a sincere manner as to attach all his friends firmly to him. Both Perry and Wright were men who saw things in the larger way, and they took commanding places among their associates. Perry had the better artistic and technical capacity, while Wright had the advantage as a student of the broader sciences. Both wrote much for our current literature and their writings were so expressive and of such high literary merit that we shall be richer for all time because of their works. We shall undoubtedly have followers but shall we ever have successors?

Another man who has just passed away is Dr. S. A. Freeman of Buffalo, N. Y. Dr. Freeman was not a man with a spectacular career, and never took so prominent a place in professional life as many others, and yet there are few men in the practice of dentistry who have for the past forty years kept in closer contact with the actual progress of the art and science of dentistry from its literary expression. He was a quiet and unassuming general practitioner for man years in Buffalo. But he spent much time and money in collecting old and rare books on dentistry. He was one of the best posted men in our country on dental literature, and probably no man except possibly, Wm. H. Trueman, of Philadelphia, had a better knowledge of our out of print literature, Many of our best libraries are under obligations to Dr. Freeman for invaluable contributions in books and advice in the completion of their journal files.

Here were three modest, yet accomplished and earnest minded men, who never made themselves conspicuous or notorious by professional trickery and politics; but by earnest and helpful endeavors each in his own way, made such impressions as may well be worth our while to contemplate. It would be most inspiring to have the true or inside historical biography of such men, which could be placed in the hands of every dental graduate in our land, as he takes up the work of his life. "The very names of such men all remind us, we can make our lives sublime."

THE 1912 DENTAL JOURNALS. A new candidate for professional favor is the *New Jersey Dental Journal*; first issue January 1, 1912. This seems to be issued as the official organ of the New Jersey State Dental Society and prints a full page cut of the four principal officers of that society, besides a long list of committees, some twenty-three in number, and two of these committees contain over twenty members in each one. The faces and names are all new and unfamiliar, and we are wondering if they didn't get the wrong name on the title page. Where are Meeker, Stockton, Watkins, Sutphen and others of the old guard? The new journal publishes its creed in its salutatory which reads like an orthodox statement. The journal is modest in size but very creditably printed with good style type on good paper, and has an air of permanence and good financial support. We wish it success.

The *Western Dental Journal* comes to us with a new "make up," which, to be truthful we don't admire, largely because it inserts advertising matter throughout the reading pages, "a la" *Oral Hygiene,* only worse. We are happy, however, to find Joseph P. Root's name as editor on the front cover page. This is some compensation. He promises to quit publishing long prosy articles with superfluous adjectives and adverbs and will boil down and condense into tablet form everything that appears in his journal in the future. We are sorry to have him cut out the adjectives as they give life and flavor to literary productions, nothing will be sweet, or pretty, or lovely to him any more. Joe, you are getting old and cross, and we shall all be sorry to miss your merry talk of old times; please keep the adjectives.

The *Dental Digest* editor has also decided to quit putting pictures of fishing parties, turkeys, christmas trees and such sentimental reminders on his cover page and has agreed to run a two tone quaker design for three months unless his readers enter too vigorous a protest. We are wondering if he isn't some jealous of Hunt's juveniles? Don't give up your colors George, we are getting used to them.

Speaking of covers we are tempted to make a trip of 300 miles to have it out with our own printers, we arn't partial to smoke.

